# NURSING HOME INFECTION PREVENTIONISTS: THEIR ROLE DURING COVID-19

**DOI:** 10.1093/geroni/igae098.0514

**Published:** 2024-12-31

**Authors:** Amy Vogelsmeier, Lori Popejoy, Steven Miller, Lisa Young, Alisha Johnson, Roy Thompson

**Affiliations:** University of Missouri, Columbia, Missouri, United States; University of Missouri, Columbia, Missouri, United States; University of Missouri, Columbia, Missouri, United States; University of Missouri, Columbia, Missouri, United States; University of Missouri, Columbia, Missouri, United States; Hunter College, City University of New York, Philadelphia, Pennsylvania, United States

## Abstract

While U.S. nursing homes were required to employ infection preventionist (IP) prior to the COVID-19 pandemic, little is known about their role during the pandemic. Leaders and IPs were interviewed about IP duties as part of a larger study conducted in 24 Midwestern nursing homes to understand nursing homes’ response to COVID-19. Homes were purposively selected for the larger study to assure diverse pandemic experiences. Interviews were analyzed by four PhD researchers, doctoral student, and project coordinator using qualitative content analysis. IPs included registered nurses (RN) (16, 66%), licensed practical nurses (LPN) (7, 30%) and respiratory therapist (1, 4%) and had varied education levels including some college (6, 25%), associate’s (13, 54%) bachelor’s (3,13%) and master’s (2, 8%) degrees. Most homes (n=22; 92%) assigned IP duties to existing clinical roles e.g., directors/assistant directors of nursing, supervisors/charge nurses. COVID-related duties included reporting COVID testing to external agencies, administering vaccinations, and monitoring/training PPE use. A few, primarily RNs, also performed traditional IP duties including surveillance of residents/staff symptoms, tracking/trending unit outbreaks, implementing strategies to reduce spread, and collaborating with medical providers. Mandated reporting, a high-stakes, high-burden activity, was often shared with administrators and corporate leaders. Despite IP training, LPNs more often described not understanding their role beyond tasks. Most IPs discussed other assigned clinical roles as taking priority over their IP responsibilities. The IP role has important implications for infection prevention and management in nursing homes, however licensure differences, role clarity, and competing demands may affect the IP’s influence in this setting.

